# Stress Factors, Stress Levels, and Coping Mechanisms among University Students

**DOI:** 10.1155/2023/2026971

**Published:** 2023-06-29

**Authors:** Abdullah Alkhawaldeh, Omar Al Omari, Samir Al Aldawi, Iman Al Hashmi, Cherry Ann Ballad, Amal Ibrahim, Sulaiman Al Sabei, Arwa Alsaraireh, Mohammad Al Qadire, Mohammed ALBashtawy

**Affiliations:** ^1^Department of Community and Mental Health, Princess Salma Faculty of Nursing, Al al-Bayt University, Mafraq, Jordan; ^2^College of Nursing, Sultan Qaboos University, Muscat, Oman; ^3^College of Medicine, Sultan Qaboos University, Muscat, Oman; ^4^Health Work Committees Association, Ramallah, State of Palestine; ^5^Faculty of Nursing, Mutah University, Al-Karak, Jordan; ^6^Princess Salma Faculty of Nursing, Al al-Bayt University, Mafraq, Jordan

## Abstract

**Aims:**

To explore university students' levels of stress, stressors, and their coping style.

**Methods:**

A cross-sectional correlational design with a convenience sample (*n* = 676) of university students who completed the Student-Life Stress Inventory (SSI) and Coping Strategies Indicator (CSI) was used. *Findings*. Overall, two-thirds of the participant reported moderate levels of stress. Students with chronic illness, living alone, low CGPA, and having exams today experienced a statistically higher mean level of stress. Students who are living alone used the “avoidance” method more significantly and the “social support” method significantly less compared with students who are living with their families and friends.

**Conclusion:**

This study concurs with others that university students are prone to distress. To our knowledge, this is the first study in the region to explore the students' coping skills. Some of the employed coping and associated factors could be used to lay the groundwork for evidence-based prevention and mitigation.

## 1. Introduction

Mounting evidence supports the belief that university students experience moderate to high levels of stress [1–5]. The interplay between biology and the environment renders vulnerable adolescents and young adults to experience stress and distress. Researchers have identified various predictors of stress among university students, including individual, environmental, and coping factors. Personality traits are an example of individual factors. A study by Rettew et al. [6] found that students with high levels of neuroticism, a personality trait characterized by anxiety and negative thinking, reported higher levels of stress. In addition, students who are introverted and have a tendency to withdraw from social situations may be more susceptible to stress than those who are extroverted and enjoy social interaction [7]. There is evidence to suggest that other personality traits, such as conscientiousness and agreeableness, may also be positively associated with stress levels among university students [6].

Environmental factors such as specialisation, living situation, social support, and campus resources are also important explanatory variables of stress among university students. For example, a study by McLean et al. [8] found that students who reported lower levels of social support had higher levels of stress. In addition, students who have supportive peers and family members are more likely to have positive attitudes toward university and lower levels of stress [9]. Similarly, a meta-analysis found that students who had access to mental health services and other campus resources reported lower levels of stress than those who lacked such support [10]. Additionally, students who have access to other campus resources, such as academic support services, may be better equipped to manage stress and succeed academically [10]. Other factors which were significantly associated with a higher level of stress were smart phone use and sleeping hours [11].

Coping styles are another important predictor of stress among university students. Coping styles refer to the strategies that individuals use to manage stress and negative emotions [12, 13]. Research has shown that students who use avoidance coping strategies, such as substance abuse or denial, are more likely to experience stress than those who use positive coping strategies, such as seeking social support or engaging in physical activity [14–16]. This suggests that the way students cope with stress can have a significant impact on their stress levels and overall well-being. Amirkhan [17] conducted a factor analysis of the strategies people employ during stress and identified three strategies, known as Amirkhan's tripartite model of coping strategies [18]. The first is to seek support, the second is to try to solve the problem, and the third is to try to avoid facing the problem. Studies have been undertaken to examine the heuristic value of these coping strategies in different populations of students around the world [19, 20]. While stress has been widely documented, there is a dearth of studies on the tripartite model among students in tertiary education. The presence of stress and distress can affect the lives of students extensively if not carefully managed, as it can lead to poorer quality of life and overall life dissatisfaction [21]. It also leads to mental health problems, such as sleep disturbances [22], headaches [23], and depression [11]. A systematic review found that students who reported higher levels of stress had lower grades and were more likely to miss classes [24].

While studies examining this topic are abundant across the globe, the inconsistent findings warrant the need for further investigation, particularly since there were very few studies on this topic in the Arabian Gulf region. The 54 million-strong native population of the Arabian Gulf countries is passing through a second stage of “demographic transition” characterized by a preponderance of youth in the population [25], and the region has been labelled as “home to one of the youngest populations in the world” [26], with the majority below 25 years of age [27]. Furthermore, it is not clear how emerging changes to the academic platform, brought about by the recent pandemic, recession, and economic challenges experienced worldwide, have affected the well-being of students in tertiary education.

### 1.1. Purpose

The study explores university students' stress, stressors, and coping styles, with the aim of resolving the following: (i) level of stress among university students, (ii) types of stressors they face, (iii) their coping mechanisms, and (iv) the relationships between the level of stress and some academic, health-related, and sociodemographic variables.

## 2. Methods

A descriptive cross-sectional design was used to gather the data from students at the only national university in Oman, Sultan Qaboos University (SQU). SQU has a student intake from all parts and strata of society, with nine colleges: Agricultural and Marine Sciences, Art and Social Sciences, Economics and Political Science, Education, Engineering, Law, Nursing, Medicine and Health Science, and Science. A random sampling technique was used. Data were gathered using an online questionnaire. Eligible participants were students who met the following inclusion criteria: (a) registered in the university and (b) can read and understand English. Failure to respond to more than 20% of the items was one of the exclusion criteria. The sample size was calculated using Slovin's formula (*n* = *N*/(1 + *N e*2), where *n* = number of participants, *N* = total population, and *e* = margin of error (0.05). The university has 16,000 students; hence, the sample size will be 400 participants.

### 2.1. Data Collection

Researchers contacted the Public Relations Department in order to send random e-mails to potential participants who met the inclusion criteria. Public Relations Department has access to all students' e-mail addresses and the required information. The e-mail contained information about the study's objectives and the researchers' contact information to inform them about their willingness to take part in the study. Then, the researchers sent an e-mail including a link to the consent form and self-reported questionnaires to complete. The survey was open between December 10 and 20, 2021.

### 2.2. Outcome Measures

The outcome measures consisted of structured questionnaires, the Student-Life Stress Inventory (SSI) and Coping Strategies Indicator (CSI), together with the participants' sociodemographic details as well as college factors. These three aspects of the study survey are detailed as follows.

### 2.3. Participants' Sociodemographics

Sociodemographic data included age, gender, current marital status, place of living, academic year, cumulative grade point average (CGPA) (to evaluate students' academic performance, students are assigned grades based on their achievement in each subject. They are graded with letters A, B, C, D, E, or F. The CGPA is derived by taking the average of all subject grade points [28]), cumulative hours (credit hours completed), work status, father's educational level, mother's educational level, monthly family income, the number of family members, the presence of chronic illness, and daily hours of sleep.

### 2.4. Student-Life Stress Inventory (SSI)

The SSI is a 40-item self-reported survey that assesses student stress. The scale is composed of four subscales: physical (e.g., headaches), interpersonal relationships (e.g., “My friends did not care about me”), academic (e.g., “I lost interest in courses”), and environmental (e.g. “Messy living conditions distracted me”). Each subscale consisted of 10 items and responses ranging from 1 to 4 (1 = never, 2 = somewhat frequent, 3 = frequent, and 4 = always) [29]. By summing the responses for the respective items, scores were calculated for the entire instrument, each part (stressors and reactions to stressors), and each category. The response scores ranged from 40 to 160, with higher scores indicating more stress. The original SSI had a strong internal consistency with Cronbach's *α* = 85.0 and an acceptable concurrent validity [30]; in the current study, Cronbach's *α* = 89.0.

### 2.5. Coping Strategies Indicator (CSI)

The Coping Strategies Indicator is a 33-item scale developed by Amirkhan [17] to assess the coping strategies that persons use to overcome their difficulties. It consists of three subscales, each consisting of 11 items: problem-solving (11 items), seeking social support (11 items), and avoidance (11 items). The responses ranged from 1 to 3 (1 = a lot, 2 = a little, and 3 = not at all). The CSI had a strong internal consistency with Cronbach's *α* = 0.84.

### 2.6. Data Analysis Plan

Data were exported to SPSS version 23 after being transformed from Google Forms to an Excel sheet. There were no missing data to treat. Descriptive statistics were used to establish the frequency, mean, and standard deviation, as well as to describe sample demographics. Independent sample *t*-tests were used to test the differences in the mean between the dependent continuous variables SSI and CSI and the independent variables which consisted of two levels such as gender, place of living, and having a chronic illness. The one-way analyses of variance (ANOVA) were used to test the differences in mean between the dependent continuous variables SSI and CSI and the independent variables which consisted of three levels including family income, CGPA, and with whom you are living. Multiple linear regression was also run to test the association between stress and the rest of the variables. An alpha level of *p* < 0.05 was set for significance in all analyses.

### 2.7. Ethical Considerations

Ethical approval was obtained from the Research and Ethics Committee of the university before the data collection began (CON/DF/2021/1). The Helsinki Declaration [31] guided our methods during the research study. Participants were informed that participation was voluntary and that they could withdraw at any time or refuse to answer any questions. Participants' confidentiality was maintained because no personal information was collected.

## 3. Results

### 3.1. Demographic Profile

Six hundred and seventy-six university students with an average age of 20.7 (SD = 2.6) completed the survey. Almost two-thirds (*n* = 463; 68.5%) were female, one-third (*n* = 204; 30.2%) achieved a cumulative GPA of 2.75–3.29, and the majority were single (*n* = 929; 93%) and with no history of chronic illness (*n* = 605; 89.5%). Almost three-quarters of the students suffered from moderate stress (*n* = 508; 75.1%), 13.5% (*n* = 91) reported severe stressors, and only 11.4% (*n* = 77) reported mild stress (Table 1).

### 3.2. Prevalence of Stress and Coping Methods Used by University Students

There were four different sources of stress: physical, academic, environmental, and interpersonal relationships. Just over half of the students experienced moderate levels of physical (*n* = 367; 54.3%) and environmental (*n* = 353; 52.2%) stressors. Over two-fifths experienced severe forms of academic (*n* = 302, 44.7%) stressors, and over two-thirds experienced severe forms of environmental (*n* = 249; 36.8%) stressors. As a response to these stressors, almost two-thirds of the participants (*n* = 509; 75.3%) used problem-solving methods, more than half (*n* = 417; 61.7%) used social support methods, and just over a third used (*n* = 256; 37.9) avoidance coping methods (Table 2).

### 3.3. Bivariate Analysis of SSI

A comparison between the total means of different SSI items and student characteristics was conducted to investigate whether there were any factors associated with SSI. The results of the *t*-test and one-way ANOVA showed that students with chronic illness experienced a statistically higher mean level of stress than their counterparts who do not have a chronic illness (*t* (674) = 3.173; *p*=0.003). Students who are living alone (*F* (2, 673) = 5.643; *p*=0.004) and having exams today (*t* (674) = 2.464; *p*=0.014) have a higher mean level of stress. One-way ANOVA showed a significant difference in the mean distribution of the SSI between participants who adopted social support as a coping method (*F* (3, 672) = 13.76; *p* < 0.01). *Post hoc* tests showed that the mean stress level among students who used social support at a “very low” level was significantly higher than that among the rest of the groups (*p* < 0.05). The distribution of the mean scores of SSI among students who used “very low” and “low” problem-solving skills was significantly higher than that of “average” and “high” users (*F* (3, 672) = 10.01; *p* < 0.01). The average SSI was significantly higher among those who were “high” users of avoidance (*F* (3, 670) = 27.7; *p* < 0.01). Overall, there was a significant positive association between the number of siblings (*r* (674) = 0.106; *p*=0.006) and the number of hours using the smartphone (*r* (674) = 0.135; *p* < 0.01) from one side and SSI from another side (Table 3).

### 3.4. Bivariate Analysis of Coping Methods

A comparison between the total mean of different coping methods' scores and student characteristics was conducted to investigate whether there were any factors associated with students' different coping methods. The results of the *t*-test and one-way ANOVA showed no significant difference in the scores for different coping methods between gender, marital status, place of living, CGPA, the number of credit hours completed, and income. However, students who are living alone used the “avoidance” strategy more significantly (*F* (2, 671) = 5.794; *p*=0.003) and the “social support” method significantly less (*F* (2, 673) = 4.89; *p*=0.008) compared with students who are living with their families and friends. Interestingly, students with chronic illness were significantly less likely to use the “social support” method (*t* (674) = 2.16, *p*=0.031) compared with their healthy counterparts, and students with low income used the “avoidance” strategy significantly less than those with high income (*F* (3, 672) = 4.712; *p*=0.003). Interestingly, there was a significant mean difference among students in different colleges regarding problem-solving (*F* (8, 625) = 3.09; *p*=0.002) and social support (*F* (8, 625) = 2.33; *p*=0.018) coping methods. *Post hoc* tests showed that students at the Colleges of Medicine, Science, and Economics used problem-solving methods significantly more than those at the rest of the colleges. Students at the College of Nursing used social support as a coping method more than their counterparts in the other colleges (Table 4).

Multiple linear regression was used to test the association between the outcome variable stress and rest of the explanatory variables. Dummy variables were created for categorical variables with more than two levels. The final model was significant compared with the constant (*F* (12, 663) = 15.1; *p* < 0.01). Significant associated factors of stress were the number of siblings, having chronic illness, cumulative hours completed, having an exam today or tomorrow, income, being in the Nursing or Agriculture College, problem-solving, avoidance, and social support coping. *R*^2^ and adjusted *R*^2^ of the final model were 0.215 and 0.20, respectively (Table 5).

## 4. Discussion

The current study explored university students' levels of stress, stressors, and their coping styles. The results revealed that the majority of students have a moderate level of stress (75.1%), followed by those with a severe level of stress (13.5%) and a mild level of stress (11.4%). Stress and burnout syndrome have emerged in the Arabian Gulf countries from research using different measures such as Influence of Studying on Student Health (ISSH), Perceived Stress Scale (PSS), Stress subscale of Depression Anxiety Stress Scales (DASS-21), Copenhagen Burnout Inventory, Cohen's Perceived Stress Scale, Common Stressor Inventory, Maslach Burnout Inventory, and Maslach Burnout Inventory (Student Survey). In Oman, in studies of medical students, various researchers including Aboalshamat et al. [32], Al-Alawi et al. [33], Al-Dabal et al. [34], Al-Khani et al. [35], Al Rasheed et al. [36], and Mahfouz et al. [37] have reported the rate of stress and distress as ranging from 7.4% to 96.3%. In Bahrain, Al Ubaidi et al. [38] and Sanad [39] found 47% and 92% to be subject to stress and distress, respectively; in Qatar, Fadhel and Adawi [40] reported 89.2% stress and distress. In Kuwait, the prevalence of 43% and 43.8% was reported by Ahmed et al. [41] and Badr et al. [42], respectively. The rate in the present study appears to tend towards a higher figure, even though such comparison may not be valid because of the use of different assessment measures and catchment areas.

The different stressors among study participants were also assessed, and the largest groups were found to have a severe level of environmental (36.8%) and academic (44.7%) sources of stress. Most of the undergraduates come from primarily rural areas in Oman, where they have studied for five to six years. Thus, they find themselves in an unfamiliar environment, with stressors that are common in a capital city such as traffic, construction work, and loud noises. Also, students moving to a new environment need to adjust to cultural disparities among their classmates, differences in socioeconomic status, and physical appearance, which all count as stressors. Furthermore, the majority of the participants reported that they had an exam on the data collection day or the day after, which might explain the high prevalence of severe academic sources of stress among the study groups. This finding is consistent with that reported in other studies, demonstrating that stress from academic work and its related responsibilities were the leading types among university students [43, 44]. In comparison, a recent study conducted among medical undergraduate students in Malaysia found that worries about the future and financial difficulties were the common stressors [45]. However, among our cohort of students, tuition fees and some living expenses are covered for all Omani citizens studying at SQU, which might explain why financial difficulties were not reported among the top stressors. Nevertheless, this finding suggests that adequate support services should be provided for university students.

Interestingly, students in the Colleges of Medicine, Science, and Economics used problem-solving methods significantly more than those at other colleges. Students at the College of Nursing used social support as a coping method more than their counterparts in the other colleges. Students' preferences concerning the methods for managing stress vary according to their field of study [46]. According to WHO/EHA policy, coping methods have no set criteria; rather, they vary based on sociocultural factors such as geography, social groups, gender, age, and historical period and are heavily influenced by a person's prior experience [47]. In the current study, we found that the most widely employed coping mechanisms were problem-solving and social support. These findings were consistent with a past experimental study that indicated that it is strongly advised to encourage the students' use of social support as coping mechanisms [48]. Among the Ghanaian and Malaysian university students, active coping, restrain coping, religious coping reframing, and planning were the most frequently reported strategies [45, 49]. The majority of the students in previous studies were found to use social support and problem-solving as coping strategies more than other strategies [44, 50]. These results point to the necessity for stress-management training programs or workshops tailored to university students' needs, cultures, and religions. Given the negative consequences of stress on an individual's health and academic performance, policymakers in higher education should consider including such training programmes for incoming students.

## 5. Strength and Limitations

The major strength of the current study was its sample size and sampling technique, which strengthened the statistical power and decreased the possibility of type 2 error. For more accurate results that are more representative of the population, we used a sample that was larger than the one that was estimated. The use of sample size calculations has a direct impact on research outcomes. Small sample sizes jeopardize a study's internal and external validity. Small differences in very large samples tend to be transformed into statistically significant differences, even when they are clinically unimportant [51]. However, this study has various flaws that should be addressed in future research. One of these is the sample composition, which varied in terms of gender representation and degree type. Therefore, the study findings should be generalized with caution. Also, future studies should employ more extensive recruitment processes to ensure more gender- and degree type-balanced populations [52]. Replicating this study with a larger, diverse, and randomized sample might broaden the body of knowledge of stress among university students.

Based on our findings, we agree with the results of previous studies highlighting the importance of stress prevention by increasing the student's awareness of effective coping strategies. The College of Nursing can play a role through starting campaigns to teach students effective coping strategies and life skills. They need also to amalgamate topics related to stress and coping in elective courses which discuss health issues.

In conclusion, while this is not the first study to report stress and distress among students in tertiary education, to date, it is the first to employ the Student-Life Stress Inventory in Oman. The most original part of this study is that it reveals the coping strategies of the students. Deciphering whether they employ adaptive or maladaptive coping will make it possible to consider corrective measures. Similarly, the associated factors identified could also contribute to the prevention and mitigation of stress and distress among students in tertiary education.

## Figures and Tables

**Table 1:** Sample's characteristics.

Variable	*n*	%
*Gender*		
Male	208	30.8
Female	468	69.2
*Place of living*		
On-campus	333	49.3
Off-campus	343	50.7
*CGPA*		
<2.0	41	6.10
2.00–2.29	76	11.2
2.30–2.74	174	25.7
2.75–3.29	204	30.2
3.30–3.74	110	16.3
3.75–4.00	26	3.80
*SSI*		
Mild stress	77	11.4
Moderate stress	580	75.1
Severe stress	91	13.5
*College*		
Agriculture	50	7.40
Arts	79	11.7
Education	59	8.70
Engineering	58	8.60
Law	51	7.50
Nursing	134	19.8
Medicine	53	7.80
Science	79	11.8
Economics	71	10.5
*Credits completed*		
≤50	337	79.9
>50	339	50.1
*Marital status*		
Married	44	6.50
Single	632	93.5
*Chronic illness*		
Yes	62	9.20
No	614	90.8
*Exam today*		
Yes	323	47.8
No	353	52.2
*With whom living*		
Alone	81	12.0
Family	293	43.3
Friends	302	44.7
*Family income*		
<1000	283	41.9
1000–1499	190	28.1
1500–1999	98	14.5
	*M*	*SD*
*Age*	20.7	2.60
*Sleep every night*	6.32	1.36
*Smartphone use*	5.6	2.60
*SSI*	102	17.6
*SSI-physical*	22.8	5.80
*SSI-interpersonal relationship*	23.8	5.00
*SSI-academic*	28.1	6.40
*SSI-environmental*	27.3	6.30
*Number of siblings*	6.41	2.93
*CSI-problem-solving*	25.3	4.50
*CSI-avoidance*	23.4	3.40
*CSI-social support*	22.1	5.30

^*∗*^CSI = Coping Strategies Indicator; ^*∗*^SSI = Student-Life Stress Inventory.

**Table 2:** Sources of stress and coping methods used by university students.

Variable	*n*	%
*SSI-physical*		
Mild	224	33.1
Moderate	367	54.3
Severe	85	12.6
*SSI-academic*		
Mild	67	9.9
Moderate	307	45.4
Severe	302	44.7
*CSI-problem-solving*		
Very low	24	3.60
Low	96	14.2
Average	509	75.3
High	47	7.00
*CSI-avoidance*		
Very low	11	1.60
Low	331	49.0
Average	256	37.9
High	76	11.2
*SSI-interpersonal relationship*		
Mild	138	20.4
Moderate	447	66.1
Severe	91	13.5
*SSI-environmental*		
Mild	74	10.9
Moderate	353	52.2
Severe	249	36.8
*CSI-social support*		
Very low	43	6.4
Low	141	20.9
Average	417	61.7
High	75	11.1

^*∗*^CSI = Coping Strategies Indicator; ^*∗*^SSI = Student-Life Stress Inventory.

**Table 3:** Bivariate analysis of SSI.

Variable	*M*	SD	*P*
*Gender*			
Male	100.1	16.81	0.059
Female	102.8	17.88	
*Chronic illness*			
Yes	108.68	18.39	**0.003**
No	101.23	17.39	
*Place of living*			
On-campus	102.98	17.77	0.138
Off-campus	100.97	17.40	
*With whom living*			
Alone	108.01	17.85	**0.004**
Family	101.51	18.30	
Friends	100.78	16.54	
*CGPA*			
Less than 2.0	104.30	17.21	**0.002**
2.002–2.29	103.72	16.80	
2.30–2.74	105.40	17.85	
2.75–3.29	101.84	17.23	
3.30–3.74	99.85	18.28	
3.75–4.00	91.34	13.10	
*Family income*			
<1000	104.00	17.81	0.051
1000–1499	100.66	17.04	
1500–1999	101.71	18.37	
>2000	99.05	16.85	
*CSI-social support*			
Very low	116.49	18.32	**<0.01**
Low	104.13	18.08	
Average	99.60	17.16	
High	102.68	14.03	
*Marital status*			
Married	97.2	17.45	0.066
Single	102.3	17.57	
*Exam today*			
Yes	103.70	17.37	**0.014**
No	100.37	17.67	
*Credits completed*			
≤50	102.81	18.15	0.209
>50	101.15	17.01	
*College*			
Agriculture	101.38	16.50	**<0.01**
Arts	106.14	19.60	
Education	102.49	17.80	
Engineering	105.10	14.14	
Law	105.30	14.14	
Nursing	95.25	17.50	
Medicine	97.81	16.10	
Science	103.25	18.60	
Economics	106.56	18.91	
*CSI-problem-solving*			
Very low	116.00	20.53	**<0.01**
Low	106.64	16.00	
Average	100.96	17.34	
High	96.10	17.04	
*CSI-avoidance*			
Very low	104.10	28.67	**<0.01**
Low	96.73	16.19	
Average	104.80	16.84	
High	114.43	15.63	

*Age*			0.146
*Sleep every night (hours)*			**0.005**
*CSI-problem-solving*			**<0.01**
*CSI-avoidance*			**<0.01**
*Number of siblings*			**0.006**
*Smartphone use (hours)*			**<0.01**
*CSI-social support*			**<0.01**

^*∗*^CSI = Coping Strategies Indicator; ^*∗*^SSI = Student-Life Stress Inventory. Bold values denote the statistical significance at the *p* < 0.05 level.

**Table 4:** Bivariate analysis of avoidance, problem-solving, and social support among university students.

Variable	Avoidance			Problem-solving			Social support		
	*M*	SD	*P*	*M*	SD	*P*	*M*	SD	*P*
*Gender*									
Male	23.20	3.57	0.387	25.48	4.75	0.484	22.01	5.37	0.863
Female	23.43	3.29		25.22	4.29		22.10	5.28	
*Marital status*									
Married	23.38	3.16	0.96	26.22	3.95	0.15	23.15	4.68	0.16
Single	23.36	3.39		25.24	4.47		22.00	5.33	
*Place of living*									
On-campus	23.20	3.34	0.226	25.05	4.47	0.141	22.14	5.29	0.553
Off-campus	23.51	3.34		25.57	4.43		21.92	5.30	
*With whom living*									
Alone	24.08	3.16	**0.003**	25.12	4.48	0.563	20.50	4.71	**0.008**
Family	23.63	3.25		25.14	4.25		22.00	5.27	
Friends	22.90	3.53		25.57	4.67		22.57	5.39	
*CGPA*									
Less than 2.0	23.51	3.22	0.930	25.4	4.43	0.063	22.97	5.92	0.546
2.002–2.29	23.64	3.53		25.2	3.84		22.23	9.57	
2.30–2.74	23.41	3.60		24.59	5.10		21.51	5.34	
2.75–3.29	23.23	3.20		25.33	4.22		22.09	5.28	
3.30–3.74	23.55	3.50		25.69	4.04		22.47	5.41	
3.75–4.00	23.73	3.50		27.26	4.16		22.65	4.56	
*Chronic illness*									
Yes	23.77	3.89	0.312	24.47	5.26	0.119	20.69	5.07	**0.031**
No	23.31	3.33		25.39	4.35		22.21	5.32	
*Exam today or tomorrow*									
Yes	23.41	3.53	0.692	25.18	4.40	0.437	21.78	5.36	0.157
No	23.31	3.25		25.43	4.47		22.34	5.26	
*Family income*									
<1000	22.87	3.22	**0.003**	25.12	4.31	0.744	21.61	5.39	0.195
1000–1499	23.86	3.30		25.63	4.2		22.50	5.13	
1500–1999	23.35	3.63		25.63	5.01		21.80	5.66	
>2000	23.89	3.45		24.92	4.69		22.67	5.07	
*College*									
Agriculture	22.86	3.35	0.573	24.44	4.88	**<0.01**	20.88	5.22	**0.005**
Arts	23.37	3.72		24.67	5.38		22.48	5.95	
Education	23.16	3.56		24.96	3.63		21.3	5.89	
Engineering	23.44	3.41		24.48	4.55		21.17	5.19	
Law	23.84	3.10		25.21	4.40		22.23	5.17	
Nursing	23.10	3.44		24.78	3.85		23.49	4.20	
Medicine	23.54	3.28		27.75	3.42		21.17	5.19	
Science	23.33	3.14		25.59	4.56		21.77	5.32	
Economics	24.11	3.60		25.63	4.69		21.32	5.41	
*Credits completed*									
≤50	23.33	3.42	0.81	25.44	4.55	0.41	22.22	5.51	0.47
>50	23.40	3.33		25.20	4.33		21.93	5.10	

*Age*			0.66			0.181			0.779
*Sleep every night (hours)*			0.968			0.509			0.216
*Number of siblings*			0.361			0.531			**0.012**
*Smartphone use (hours)*			0.547			0.091			0.761
*SSI-total*			**<0.01**			**<0.01**			**<0.01**
*SSI-physical*			**<0.01**			**0.001**			**<0.01**
*SSI-interpersonal relationship*			**<0.01**			**<0.01**			**<0.01**
*SSI-academic*			**<0.01**			**<0.01**			**0.01**
*SSI-environmental*			**<0.01**			0.910			0.261

^*∗*^CSI = Coping Strategies Indicator; ^*∗*^SSI = Student-Life Stress Inventory. Bold values denote the statistical significance at the *p* < 0.05 level.

**Table 5:** Factors associated with stress (SSI).

Model	Unstandardized coefficients		Standardized coefficients		
	*B*	Std. error	*β*	*t*	*p*
Constant	103.616	8.255		12.552	**<0.01**

Gender (relative to male)	1.923	1.331	0.050	1.445	0.149
Number of siblings	0.677	0.215	0.113	3.153	**0.002**
Credits completed (relative to <50)	−2.658	1.222	−0.076	−2.175	**0.030**
Chronic illness (relative to yes)	−5.607	2.119	−0.092	−2.646	**0.008**
Having exams (relative to yes)	−2.914	1.230	−0.083	−2.369	**0.018**
Sleep every night (hours)	−1.321	0.453	−0.102	−2.918	**0.004**
Daily smartphone use (hours)	0.729	0.218	0.106	3.341	**0.001**
^*∗*^CSI-problem-solving	−0.654	0.145	−0.165	−4.526	**<0.01**
^*∗*^CSI-social support	−0.308	0.122	−0.093	−2.518	**0.012**
^*∗*^CSI-avoidance	1.648	0.183	0.316	8.997	**<0.01**
Income: 1001–1500 (relative to <1000)	−3.446	1.430	−0.088	−2.410	**0.016**
Income: >2000 (relative to <1000)	−4.651	1.790	−0.096	−2.598	**0.010**

^*∗*^CSI = Coping Strategies Indicator. Bold values denote the statistical significance at the *p* < 0.05 level.

## Data Availability

The data used to support the findings of this study are available from the corresponding author upon request.
